# Mentoring program: bridging gaps for international authors

**DOI:** 10.1093/radadv/umae036

**Published:** 2025-02-15

**Authors:** Luiz Celso Hygino da Cruz, Antonio Luna

**Affiliations:** Neuroradiology, CDPI—Dasa, Hospital Pro-Cardiaco, Rio de Janeiro, RJ 22290240, Brazil; HT Medica, Clinica Las Nieves, Jaen 23006, Spain

**Keywords:** mentoring, international, publication, authors

Dear Editor,

The article by Pandey et al.^1^ highlights an important shift toward greater inclusivity in academic publishing. While the benefits for international radiologists—professional growth, mentorship, and access to a global network—are substantial, significant barriers still limit broader participation from diverse geographic and socioeconomic backgrounds.

The Radiological Society of North America (RSNA) has made a commendable effort to increase international inclusion and support for radiologists from diverse backgrounds. RSNA journals have introduced several initiatives; *RadioGraphics* has carried out different steps in this direction through the vision of the Editor and its International Team toward addressing these barriers.^2^ These international initiatives are essential, as radiology journals have a growing global readership, yet equitable access to publication opportunities and educational resources remains challenging.

One major obstacle for international authors is the limited availability of mentorship and representation, particularly for researchers from low- and middle-income countries that may lack access to mentors familiar with high-impact journal standards and limited access to experienced mentors. The *RadioGraphics* mentoring program is a pivotal initiative that supports international authors, particularly those from non-native English-speaking countries, in navigating the complex publication process.

The journey to publish an article in *RadioGraphics* begins with crafting an abstract for the RSNA Annual Meeting *Education Exhibits*. Accepted abstracts progress to conference presentations, where panelists meticulously review exhibits. Only a select few are invited to submit manuscripts to *RadioGraphics*. These manuscripts then undergo rigorous peer review, with an average of 3 reviewers.

International authors of selected *Education Exhibits* are invited to join the mentoring program. Paired with seasoned *RadioGraphics* authors, they receive guidance on structuring arguments, refining language, and meeting editorial expectations.

Mentors who are seasoned *RadioGraphics* authors dedicate significant time to providing detailed feedback, guiding manuscript development, and helping authors meet editorial standards. As mentors are included as coauthors—never as first or senior authors—this process demands substantial effort and time. This level of commitment has posed an additional challenge in recruiting mentors willing to participate in the program.

This program has achieved remarkable success since its inception during RSNA 2020, with every mentored manuscript reaching publication. It highlights how mentorship can bridge gaps, empower international authors, and enrich the journal with diverse perspectives.

In conclusion, RSNA is paving the way for a more inclusive and accessible academic publishing environment. By expanding educational opportunities and fostering international collaborations, *RadioGraphics* and its International Team have made meaningful progress in achieving this goal. These initiatives not only enhance knowledge-sharing but also strengthen ties within the global radiology community. They align with RSNA’s mission of promoting diversity and equity and have the potential to make radiology truly global.
